# Wasting and associated factors among critically ill children admitted to pediatric intensive care unit in Ethiopia

**DOI:** 10.1186/s40795-022-00506-x

**Published:** 2022-02-02

**Authors:** Nahom Worku Teshager, Ashenafi Tazebew Amare, Koku Sisay Tamirat, Melkamu Aderajew Zemene

**Affiliations:** 1 Department of Pediatrics and Child Health, School of Medicine, College of Medicine and Health Sciences, University of Gondar, Gondar, Ethiopia; 2 Department of Epidemiology and Biostatistics, Institute of Public Health, College of Medicine and Health Sciences, University of Gondar, Gondar, Ethiopia; 3 Public Health Department, College of Health Sciences, Debre Tabor University, Debre Tabor, Ethiopia

**Keywords:** Wasting, Children, Intensive care units, Ethiopia

## Abstract

**Background:**

Nutritional problems are increasingly associated with acute infections. It is also related to further complications of illnesses and poor treatment outcomes of medical conditions. This study aimed to assess wasting and associated factors among critically ill children admitted to intensive care units at the time of admission.

**Methods:**

An institution-based prospective observational study was employed among children admitted to pediatric intensive care of the University of Gondar Comprehensive Specialized Hospital from February 1, 2018, to July 30, 2019. Data about socio-demographic, clinical, and anthropometric measurements were taken from children at the time of admission and length of hospital stay and treatment-related data were collected by chart review at discharge. Summary measures were computed and presented in the form of text, tables, and graphs. A *p*-value of less than 0.2 was used to select candidate variables for multivariable analysis. A binary logistic regression model was fitted to identify factors associated with wasting. Adjusted odds ratio with 95% confidence interval (CI) was calculated and variables with a p-value less than 0.05 in the multi-variable analysis were considered to declare factors associated with wasting.

**Results:**

The median age at admission was 48 (IQR: 12 to 122) months. Of the total admitted children to ICU, 47.97% were undernourished, of which 32% (95%CI: (26.8% to 37.4%) were severely wasted. Caregivers who had no formal education (AOR=4.43, 95%CI 1.62 12.10), transferred from wards (AOR=2.98, 95%CI: 1.02 8.69), duration of illness ≥6 days before health facility visit (AOR=2.14, 95%CI: 1.22 3.72) and comorbidity (AOR=6.85, 95%CI: 2.93 16.05) were statistically significant factors associated with wasting.

**Conclusion:**

Wasting was high among children admitted to the intensive care unit. No formal education, transferred from wards and operation rooms, longer duration of illness before health facility visits, and comorbidity were factors associated with wasting. Wasted patients had higher mortality as compared to patients with no wasting. A multicenter study with larger sample size is recommended for a more generalizable result.

## Background

Childhood is the period where the major developmental milestone changes undergo greater nutritional demands [1]. However, undernutrition among children is a major public health problem, particularly in low and middle-income countries. According to the 2016 global report, about 45% of under-five mortality was associated with undernutrition [2]. Nutritional problems are increasingly associated with acute infections, further complications of illnesses, and poor treatment outcomes of medical conditions [2]. In addition, undernutrition is associated with longer negative health consequences such as developmental delay, recurrent infections, neurocognitive problems, and generational defects [3–7].

Serious medical conditions in children that deserve admission to the intensive care unit could be a complication of undernutrition or malnutrition could be the complication of the critical illness [8]. Hypoglycemia, infections, and dehydration are some of the acute complications of undernutrition that may end up in deaths unless the undernutrition is addressed together with critical care interventions [1, 5, 9, 10]. According to systematic review and meta-analysis, nutritional depletion among children admitted to the intensive care unit is associated with worse treatment outcomes [11]. Evidence from analyses of a huge data set from low and middle-income countries showed that severe acute malnutrition is associated with a nine times higher risk of mortality as compared to well-nourished children [12].

Independent studies in India, China, and Brazil showed 51.2, 51.7, and 18% of critically ill children were undernourished respectively [13–15]. On the other hand, a study from sixteen countries among children aged 1 month to 18 years admitted to PICU revealed that 17.9% were wasted [16]. Similarly, the overall prevalence of wasting among critically ill children in Nigeria was 26.6% [17].

A better understanding of the nutritional status of children in the intensive care units of low- and middle-income countries like Ethiopia where undernutrition (wasting and stunting) is a major public health problem is critical for a better outcome. Early nutritional intervention for wasted critically ill children may facilitate recovery from ICU [1, 18, 19]. Though different studies were conducted to assess nutritional status among children, there is a paucity of published data on the magnitude of wasting and factors associated with it among critically ill children admitted to ICU in Ethiopia and other low- and middle-income countries.

Therefore, this study aimed to examine the magnitude of wasting and associated factors from anthropometric data among critically ill children admitted to the PICU of a single center in Ethiopia. Findings from this study will serve as a baseline for future studies that might be done for the understanding of the magnitude, impact, and contribution of nutritional status to critical illness outcomes.

## Methods

### Study design, period, and setting

An institution-based prospective observational study was conducted among children aged 1months to 18 years admitted to the pediatric intensive care unit of the University of Gondar Comprehensive Specialized Hospital from February 1, 2018to July 30, 2019.

This is a teaching hospital with a total of 641 beds and 96 beds on the pediatrics side. The PICU has six beds; on average there are about 25 pediatric critical care admissions per month. The ICU team is composed of a general pediatrician, residents, interns, and a handful of senior-level nurses, but there are no dieticians.

### Population and sample

All children who were admitted to the pediatric intensive care unit during the data collection period were considered as the study population. Patients aged 1 month to 18 years admitted to the pediatric intensive care unit were included in the study. Patients who do not have a caregiver or whose caregivers do not have adequate information about the socio-demographic status of the patient were excluded from the study. Moreover, those patients who are terminally ill or are on mechanical ventilators were excluded from the study due to the difficulty of measuring their weight.

### Data collection procedure

Data was collected by physicians who were treating them using a standardized questionnaire after taking consent from caretakers. There were orientations and training about data collection (proper measurement of length, weight, and MUAC) and the objective of the study every three months as the ward team rotates every three months and demonstration every Monday for data collectors. Data on clinical characteristics were taken from the documentation of the admitting physician by chart review. Socio-demographic data and medical history were taken by interview. Diagnoses were put based on the WHO International Classification of Diseases 10^th^ version (ICD-10) for disease category and only the primary diagnosis was used for ICD-10 assignment in patients having multiple diagnoses [20, 21]. A nutritional assessment like weight, height/ length, the mid-upper circumference (MUAC) was taken at admission and Z-score was computed by using WHO Anthro/plus. The collected data were double-checked by the data collector and the principal investigator. The principal investigators supervised the overall process and check the completeness of questionnaires every day.

### Variable of the study and operational definitions

Acute malnutrition (wasting) was the outcome variable, whereas socio-demographic characteristics (age, sex, relation with the caregiver, caregiver’s educational status, occupation), hospital arrival, and admission related factors (duration of illness before admission, the month of admission, source of admission), clinical characteristics (diagnosis, anthropometric value at admission, comorbidity, vaccination status) were the independent variables.

#### Acute malnutrition (Wasting)

Nutritional status of a child when the weight for height/length Z-score is <-2SD as compared with the median value of World Health Organization reference point [22].

#### Length of Stay (LOS)

Refers to the duration of stay in a number of days from the date of admission to the date of discharge in PICU.

### Data processing and analysis

After the data were checked for its consistency and completeness, it was entered into EpiData version 3.1 exported to STATA version 14 for cleaning and analysis. Descriptive statistics like mean, median, proportions were carried out to summarize baseline socio-demographic and clinical characteristics. A *p*-value of less than 0.2 was used to select candidate variables for multivariable analysis. A binary logistic regression model was fitted to identify factors associated with wasting. Adjusted odds ratio with 95% confidence interval (CI) was calculated and variables with a *p*-value less than 0.05 in the multi-variable analysis were considered to declare factors associated with wasting. We used the Kaplan-Meier survival estimation curve and log-rank tests to compare the difference in LOS as well as mortality between normal and wasted patients and among normal, moderately wasted, and severely wasted patients.

## Result

### Socio-demographic characteristics

A total of 376 patients were admitted during the study period. Data was collected from 327 patients who fulfilled the inclusion criteria. Fourteen patients were excluded from the study for having incomplete data. Three hundred thirteen participants were included in the final analysis.

The median age at admission was 48 (IQR: 12 to 122) months, about 28.1% were infants followed by adolescents (21.4%).

### Clinical characteristics of children admitted to pediatric intensive care units

The major reason for PICU admission was summarized based on the 10^th^ version of the ICD of WHO. Neurologic disorders (22.7%), infectious disease (18.8%), and environmental hazards (11.8%) account for the top three diagnoses. The median duration of stay in the ICU was 3 days with IQR: 1-6 days. Forty-three (13.7%) patients had at least one comorbid illness, of which congenital malformations and genetic disorders (27.9%), cerebral palsy with or without seizure disorders (25.6%), CKD (16.3%), and HIV/AIDS (14%) are the commonest illnesses (Table 1).

**Table 1:** Socio-demographic and clinical characteristics of children admitted to the pediatric intensive care unit at the University of Gondar comprehensive specialized hospital, northwest Ethiopia February 1, 2018, to July 30, 2019 (*n* = 313)

Characteristics	Frequency	Percentages (%)
**Age in months**		
≤ 12	88	28.1
13-24	29	9.3
25-60	66	21.1
61-132	63	20.1
>132	67	21.4
**Sex**		
Male	187	59.7
Female	126	40.3
**Residence**		
Urban	71	22.7
Rural	242	73.3
**Caregivers**		
Parents	291	93
Grandparents	8	2.6
Siblings	8	2.6
Others	6	1.9
**Caregiver level of education**		
No formal education	242	77.6
Primary school	32	10.2
Secondary school	17	5.4
College and above	21	6.7
**Caregivers’ occupation**		
Farmers	223	71.2
Merchants and private	32	10.2
Government employee	31	9.9
Unemployed	27	8.6
**Season of admission**		
Summer	63	20.1
Spring	45	14.4
Winter	85	27.2
Autumn	120	38.3
**Clinical characteristics**		
**Duration of illness before PICU admission in days**		
<6 days	164	52.4
≥6 days	149	47.6
**Sources of admission**		
Home	36	11.5
Other facilities	37	11.8
Emergency room	189	60.4
Wards	51	16.3
**Vaccination status**		
Complete	203	64.9
Incomplete	110	35.1
**Comorbid illness**		
Yes	43	13.7
No	270	86.3
**ICD 10 diagnosis**		
Neurology	71	26.7
Infectious disease	59	18.8
Trauma and environmental	37	11.8
Metabolic diseases	28	8.9
Congenital malformation	23	7.4
Cardiovascular disease	21	6.7
Gastrointestinal	20	6.4
Renal diseases	20	6.4
Respiratory diseases	18	5.7
Neoplasm	18	5.7
Hematology	3	1
**Critical illness diagnosis (*n*** **= 100)**		
Sepsis	32	32
Severe sepsis	9	9
Septic shock	47	47
ARDS	12	12

### Nutritional status of children admitted to pediatric ICU

**Fig. 1:**
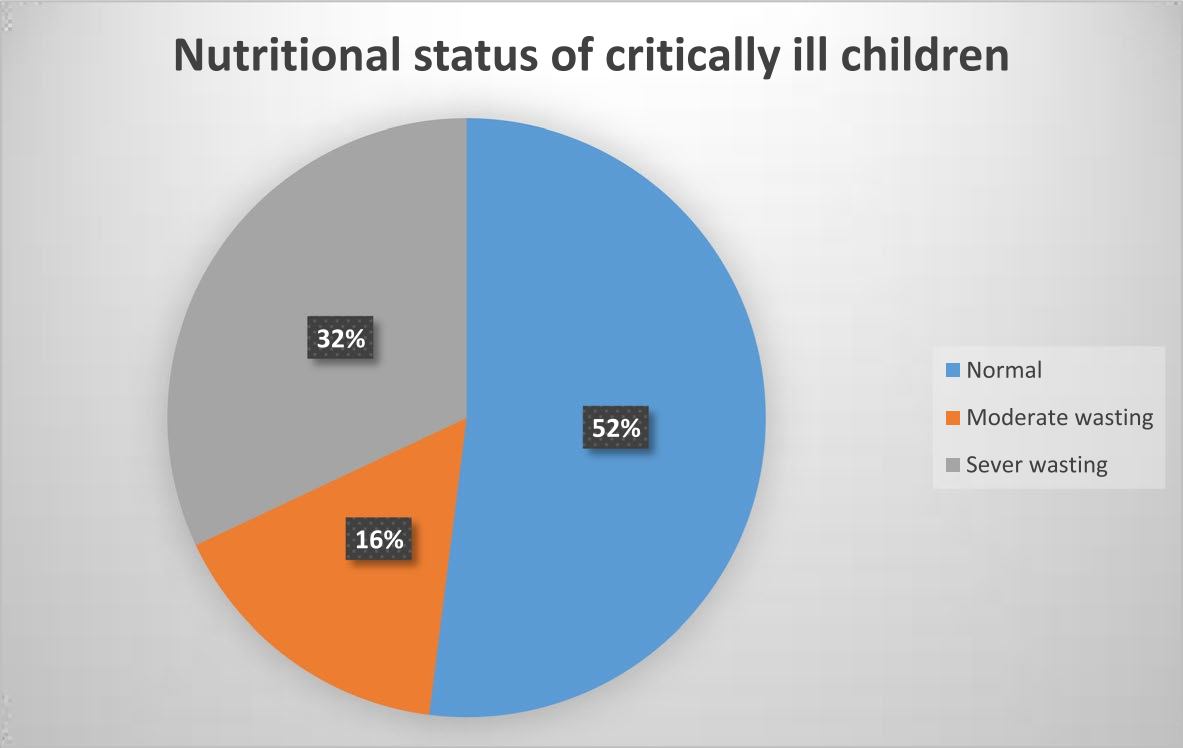
Magnitude of acute malnutrition (wasting) among children admitted to pediatric intensive care unit in Ethiopia, February 1, 2018, to July 30, 2019 (*n* = 313)

As shown in Fig. 1 below, of all participants, 48% (95% CI: 42.2, 53.6) were wasted. Among all participants, 16% were moderately wasted and 32% of them had severe wasting. Males account for 58 and 64 % of severely and moderately wasted children respectively.

### Factors associated with wasting among children admitted to ICU

From the multivariable logistic regression analysis, caregivers’ level of education, duration of illness before the visit of any health facility, comorbidities, and source of admission were significantly associated with wasting among children admitted to the pediatric intensive care unit. Thus, the odds of being wasted among children admitted to the pediatric intensive care unit whose caregiver had no formal education were 4.43 times higher as compared to those whose caregiver had primary education and above (AOR=4.43,95%CI: 1.62, 12.10). Children who were transferred from wards had 2.98 times higher odds of being wasted as compared to those coming from home (AOR=2.98, 95%CI: 1.02 8.69). Likewise, children who had comorbidities and longer than six days duration of illness before health facility visit had 6.85 (AOR=6.85, 95%CI: 2.93 16.05) and 2.14 (AOR=2.14, 95%CI: 1.22 3.77) times higher odds respectively as compared to their counterparts (Table 2).

**Table 2:** Binary logistic regression analysis to identify factors associated with wasting among children admitted to pediatric intensive care at the University of Gondar comprehensive specialized hospital, northwest Ethiopia February 1, 2018, to July 30, 2019 (*n* = 313)

Characteristics	Wasting		Crude OR	Adjusted OR
	Yes	No		
**Age in months**				
≤12	25	63	1	1
13-24	10	19	1.32(0.54 3.24)	1.16(0.41 3.31)
25-60	15	51	0.74(0.35 1.55)	0.53(0.21 1.30)
61-132	23	40	1.45(0.72 2.89)	1.15(0.50 2.63)
>132	27	40	1.70(0.86 3.33)	1.39(0.63 3.06)
**Caregiver’ level of education**				
No formal education	90	153	3.52(1.72 7.23)	4.43(1.62 12.10)*
Primary and above	10	60	1	1
**Caregivers’ occupation**				
Farmers	76	147	1	1
Merchants and private	6	26	0.44(0.17 1.15)	0.75(0.23 2.40)
Government employee	6	25	0.46(0.18 1.18)	0.64(0.15 2.81)
Unemployed	12	15	1.54(0.69 3.47)	1.58(0.59 4.24)
**Source of admission**				
Home	8	28	1	1
Other facilities	8	29	0.96(0.31 2.92)	1.08(0.32 3.59)
Emergency room	60	129	1.62(0.70 3.78)	1.88(0.75 4.73)
Wards	24	27	3.11(1.19 8.11)	2.98(1.02 8.69)*
**Duration of illness before visit any health facility**				
<6 days	38	126	1	1
≥6 days	62	87	2.36(1.45 3.84)	2.14(1.22 3.77)*
**Comorbidities**				
Yes	73	197	4.55(2.32 8.93)	6.85(2.93 16.05)*
No	27	16	1	1
**Critical illness**				
Yes	44	56	2.20(1.33 3.62)	1.48(0.82 2.65)
No	56	157	1	1

*significant at *p*<0.05

### Wasting and ICU outcome

**Fig. 2:**
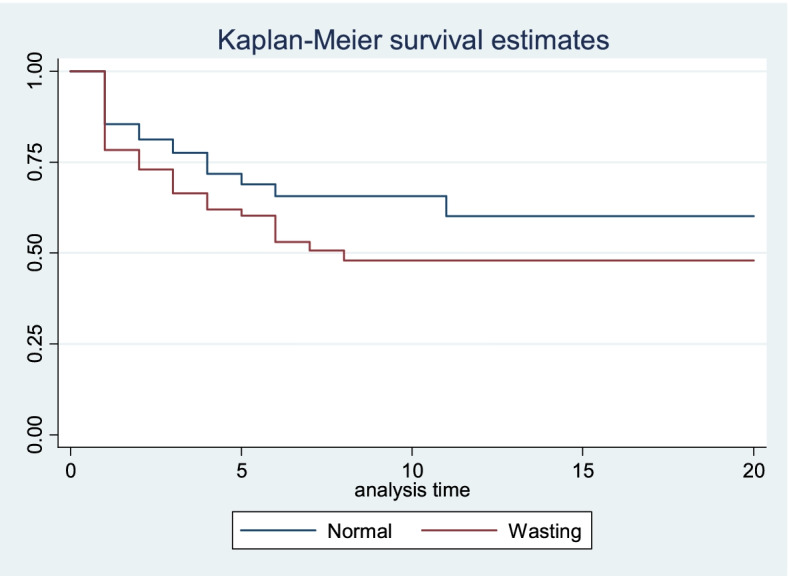
Kaplan Meier survival estimate of mortality difference between wasted and normal patients admitted to pediatric intensive care unit in Ethiopia, February 1, 2018, to July 30, 2019 (*n* = 313)

**Fig. 3:**
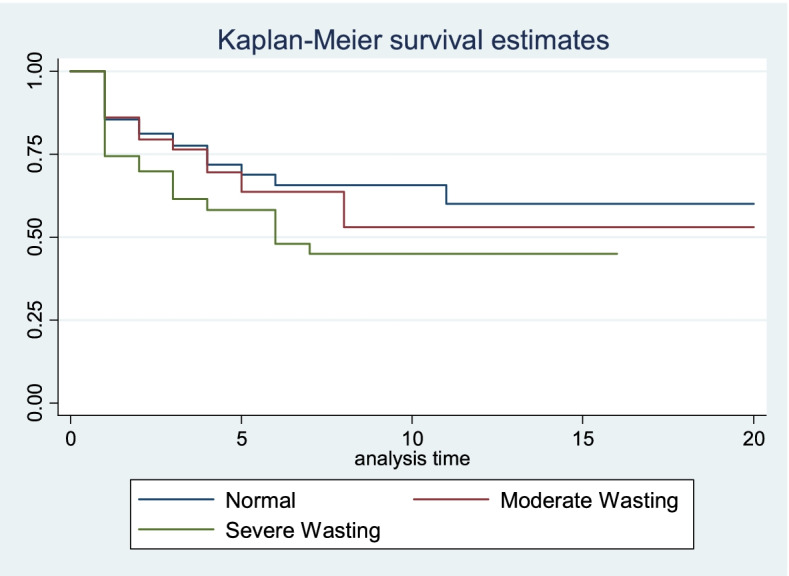
Kaplan Meier survival estimate of mortality difference among normal, moderately wasted and severely wasted patients admitted to pediatric intensive care unit in Ethiopia, February 1, 2018, to July 30, 2019 (*n* = 313)

Kaplan-Meier survival estimation and log-rank tests were used to investigate the differences in mortality and length of stay. Thus, wasted children had a statistically significant higher mortality than normal patients (x2 = 4.84, df = 1, *p* = 0.028) (Fig. 2). Furthermore, compared to normal and moderately wasted children, severely wasted children had a greater mortality rate (x2 = 6.61, df = 2, *p* = 0.037) (Fig. 3). However, there were no statistically significant differences in length of stay between normal and wasted children (x2 = 1.89, df = 1, *p* = 0.169) or between normal, moderately wasted, and severely wasted children (x2 = 2.35, df = 2, *p* = 0.308).

## Discussion

Studies on the nutritional status of children in the intensive care units of low- and middle-income countries like Ethiopia where undernutrition is a major public health problem is paramount for a better outcome. Therefore, this study aimed to assess the magnitude of wasting and associated factors among critically ill children admitted to intensive care units at the time of admission. These findings help clinicians practice nutritional status tailored care. It will also serve as a baseline for future studies.

The overall prevalence of wasting among critically ill children admitted to PICU was 48 % (95% CI: 42.2, 53.6). Of which, about 16 and 32% were moderate and severe wasting respectively. This study was consistent with findings of studies done in Brazil (43-53%) [5], India (51.2%) [13], and China (51.7%) [14]. However, this result was higher than the finding of another multi-center study done in Brazil (17.1-17.9%) [6], Iran (25.2%) [23], a study from sixteen countries (17.9%) [16], and Nigeria (26.6%) [17]. This variation could be due to socio-demographic and economic differences between the study settings. On the other hand, the finding in this study is lower than the finding of another study conducted in Brazil using serum albumin measurement (64.2%) [24]. This difference can be attributed to the difference in the nutritional assessment technique; thus, anthropometry might underestimate nutritional status.

The odds of wasting were five times higher for children whose caregivers had no formal education as compared to those who had a primary school and above. This finding was consistent with the finding of previous studies conducted in Kenya, Brazil, Nigeria, and Ethiopia [3, 8, 25, 26]. This could be because caregiver knowledge and awareness of nutritional practices affect the nutritional status of a child. Children who were transferred from wards had an increased occurrence of wasting as compared to those who were directly admitted from home. This study finding is similar to another study finding in Malawi [9]. The possible explanation might be that children transferred from wards might have hospital-acquired malnutrition. In addition, nutrition intake in the hospital might be low which could contribute to the development of in-hospital malnutrition.

Patients who had a longer duration of illness before seeking health care had two times higher odds of being wasted as compared to those who visited the health facility early. This could be related to the chronicity of the illness that leads to either increased energy requirement and expenditure or decreased intake due to the anorexic effect of inflammatory mediators (especially in infectious diseases). This finding was consistent with other studies [4, 8, 9].

Comorbidities were also associated with increased occurrence of wasting as compared to those who had no such history. This finding was in line with findings in previous studies done in different settings [25, 27, 28]. This could be because comorbid illnesses affect the intake and utilization of nutrients in the body.

Wasted children had a statistically significant higher mortality than normal patients. This finding is supported by other studies [13, 29, 30]. This could be because nutritional deficiencies potentiate the impacts of infections, organ failures, and metabolic disturbances that fuel the underlying disease process. Besides, wasted patients have limited physiologic reserve as compared to patients with no wasting.

### Strength and limitation of the study

This is the first single-center study in the country that reported the magnitude and associated factors of wasting and its impact on mortality in an intensive care setting. Nutritional status was assessed by anthropometric parameters which could underestimate the magnitude compared to biochemical measurements. It could have been good if we included dietary diversity score and household food insecurity status which have causality with the nutritional status of children. Moreover, analysis and adjustment for residual and unmeasured confounders were not done.

## Conclusion and recommendation

This study showed that the magnitude of wasting was high among children admitted to the intensive care unit. No formal education, transferred from wards, longer duration of illness before health facility visits, and comorbidity were factors associated with wasting. Furthermore, wasted patients had significantly higher mortality than normal patients. A multicenter study with larger sample size is recommended for a more generalizable result.

## Data Availability

Data is available from the corresponding author upon reasonable request.

## Abbreviations

CI: Confidence Intervals
CKD: Chronic Kidney Diseases
ICD: International Classifications of Disease
ICU: Intensive Care Unit
IQR: Interquartile Range
LOS: Length of Hospital stay
MUAC: Mid Upper Arm Circumference
PICU: Pediatric Intensive Care Unit
WHO: World Health Organization
